# Supplementary material for: Are sawfishes still present in Mozambique? A baseline ecological study
Source: PeerJ. 2017 Feb 2;5:e2950. doi: 10.7717/peerj.2950 (PMC5292025; doi:10.7717/peerj.2950)
Supplement: Appendix I [file peerj-05-2950-s001.docx]

**APPENDIX I – Interview questions**

1. ***English:***
2. Name of village:
3. Age of interviewee:
4. Years of experience (as fisherman or in his/ her role dealing with fish):
5. Type of gear used:
6. Sex:
7. Do you know what this is? (Show image of sawfish here)
8. What is the local name for this fish?
9. Have you ever seen a (sawfish) alive/ dead?

- If yes, when was the last time you saw one?
- Did you catch it yourself (the last one)?
- What type of net was used to catch it?
- Where did you see/ catch it?
- What was the length of the last sawfish you saw?

1. What did you/ they do with the sawfish?

Sell meat/ sell fins/ sell saw/ threw away/ kept to eat/ other (give details)

- If meat / fins/ saw were sold, do you remember the price they were sold for?

1. Have you seen many / did you used to catch (sawfishes) frequently?

Many per year/ 1-3 per year / 1 every 2 or 3 years / very rare

1. Are there less (sawfishes) now than in the past?

- If yes, what do you think is the cause of this change?

1. What are the cultural or religious beliefs relating to (sawfishes) in your village?
2. Do (sawfishes) have a value here?

Can you sell the meat/ fins/ saw?

- If yes, for what price?
- To whom do you sell them?

1. Do you know if anyone in the village has a sawfish rostrum we can see?
2. ***Portuguese:***

1. Qual é o nome da sua aldeia?

2. Qual a sua idade?

3. Há quantos anos é pescador?

4. Qual o tipo de redes que usa na sua pesca?

5. Sexo: Masculino ou Feminino

6. Você sabe o que é isto? (imagem de tubarão-serra, aqui)

7. Qual é o nome local para este peixe?

8. Você já alguma vez viu um tubarão-serra vivo / morto?

- Se sim, quando foi a ultima vez que você viu um?

- Foi você que o pescou?

- Qual foi o tipo de rede que usou para o pescar?

- Em que local (região/ área) é pescou o tubarão-serra?

- Qual era o tamanho desse tubarão-serra?

9. O que fez com o tubarão-serra:

Vendeu a carne/ vendeu as barbatanas/ vendeu a serra/ devolveu ao mar/ levou para comer/ outro (detalhes - )

Se vendeu a carne/barbatanas/ou a serra lembra-se a que preço é que vendeu?

10. Costuma ver muitos tubarões-serra? / Costuma pescar tubarão-serra frequentemente?

Muitos tubarões-serra por ano/ 1-3 tubarões-serra por ano/ 1 tubarão-serra a cada 2-3 anos / Muito raro apanhar um tubarão-serra.

11. Acha que existem menos tubarões-serra agora que antigamente?

Se sim, qual será a causa para haver menos tubarões-serra agora?

12. Quais são as crenças culturais ou religiosas relativas ao tubarão-serra na sua aldeia?

13. O tubarão-serra tem valor aqui?

Você pode vender a carne/ as barbatanas/ ou a serra destes animais?

Se sim, por que preço é que consegue vender?

A quem é que você os vende?

14. Alguém na sua aldeia tem uma serra de um destes animais? Se sim acha que posso ver?
