# Supplementary material for: Are sawfishes still present in Mozambique? A baseline ecological study
Source: PeerJ. 2017 Feb 2;5:e2950. doi: 10.7717/peerj.2950 (PMC5292025; doi:10.7717/peerj.2950)
Supplement: Appendix II — Historical records of sawfishes in Mozambican waters or in river systems which pass through Mozambique. Information was collated from historical texts, the International Sawfish Encounter Database and from museum specimens known to be of Mozambican origin. Each ISED record pertains to one sawfish. [file peerj-05-2950-s002.docx]

**APPENDIX II:** Historical records of sawfishes in Mozambican waters or in river systems which pass through Mozambique. Information was collated from historical texts, the International Sawfish Encounter Database and from museum specimens known to be of Mozambican origin. Each ISED record pertains to one sawfish.

| **Date** | **Species & nature of observation** | **Location** | **Length** | **Rostral tooth count** | **Source/ museum number** |
| --- | --- | --- | --- | --- | --- |
| 1858-1864 | Species unknown.  ‘*in the mouth of the river many sawfish are found…. The saw is from a foot to eighteen inches long. We never heard of anyone being wounded by this fish, nor, though it goes many hundreds of miles up the river in fresh water, could we learn that it was eaten by the people*’ | Zambezi River | no est. |  | Livingstone & Livingstone (1866) |
| Pre-1909 | *P. perotteti* (ISED)  Capture | Zambezi River | no est. |  | Boulenger (1909) |
| 1910 | Species unknown.  ‘*Several large* sawfish*, young turtle, and sharks were brought by fishermen for sale, which had been caught in reed baskets staked on the river*’ | Shire River | no est. |  | Swann (1910) |
| Pre-1950 | *P. microdon* (ISED)  Capture | Save River | no est. |  | J.L.B. Smith (1950) |
| Pre-1950 | *P. zijsron* (ISED)  Capture | Delagoa Bay | no est. |  | J.L.B. Smith (1950) |
| 1951 | *P. microdon* (ISED)  Capture - net | Save River, in fresh water (approx. position 22° S, 32° E) | no est. |  | J.L.B. Smith (1952) |
| 1953 | *P. microdon* (ISED)  Capture - net | Lourenco Marques (Maputo) | 18 ft 2 in (5.54 m) |  | Jubb (1961) |
| Pre-1961 | *P. microdon* (ISED)  Capture – reed fence | Lower Save River | ~ 9 ft (m) | 17 L, 17 R | Jubb (1961) |
| Pre-1961 | *P. microdon* (ISED)  Capture – reed fence | Lower Save River | no est. | 21 L, 22 R | Jubb (1961) |
| Pre-1967 | *P. perotteti* (ISED)  Capture | Zambezi River, approx. 60 miles from sea | 8 ft 4 in (c. 2.54 m) |  | Wallace (1967) |
| 1967 | *Pristis microdon^[[1]](#footnote-1)^*  A specimen ‘*8 ft 4 in in length was captured in the Zambesi River approximately 60 miles from the sea, where the species appears to be common*’ | Zambezi River, c. 60 mi from the sea | no est. |  | Wallace (1967) |
| 1975? | Species unknown.  ‘*sawfish are occasionally caught at the confluence of the Lundi and Sabi rivers*’^[[2]](#footnote-2)^ | Confluence of the Rundi and Save Rivers | n/a |  | Tanser (1975) |
| 1845-1846 | *P. pristis*  Collected by Wilhelm C. H. Peters | Vila de Sena, Zambezi River | 980 mm TL |  | Museum specimen. *ZMB 4527, male* |
| 1844-1846 | *P. pristis*  Collected by Wilhelm C. H. Peters | Mozambique | 1230 mm TL |  | Museum specimen. *ZMB 7851, dry male* |
| 1858-1864 | *P. pristis*  Collected by John Kirk | Zambezi River, Terre, Zambia | 360 mm (SRL) | 19 L, 17 R | Museum specimen. *BMNH 1982.9.13.7, isolated rostrum^[[3]](#footnote-3)^* |
| 1858-1864 | *P. pristis*  Collected by John Kirk | *Zambezi River, (‘Zambezi Expedition’), Mozambique* | 440 mm (SRL) | 19 L, 19 R | Faria et al. (2012)  Museum specimen. *BMNH 1864.6.28.21, isolated rostrum* |
| Poss. 20^th^ century | *P. pristis*  Collected from the Makonde tribe by Maria Luisa da Silva, 25 Oct 2007, during an ethnographic study | Mozambique | 1115 mm | 19 L, 19 R | Museum specimen *MCUC-ANT.2007.1.137* |
| Year unknown | Species unknown  Sawfish were mentioned as being present in the Save River, near Marumbene (in Zimbabwe) | Save River, near Marumbene | no est. |  | Dick-Read (2005) |

* The Lundi River (now known as the Rundi River) is a tributary of the Save (formerly known as the Sabi) River and the two join in the south-eastern corner of Zimbabwe, just before the border with Mozambique.

**References**

Boulenger 1909. Catalogue of the Fresh-water fishes of Africa in the British Museum Vol 1. Taylor and Francis, London

Dick-Read R. 2005. The phantom voyagers: evidence of Indonesian settlement in Africa in ancient times. Thurlton.

Jubb. 1961. An Illustrated Guide to the Freshwater Fishes of the Zambezi River, Lake Kariba, Pungwe, Sabi, Lundi, and Limpopo Rivers. Stuart Manning publishers.

Livingstone D, Livingstone C. 1866. Narrative of an Expendition to the Zambesi and Its Tributaries: And of the Discovery of the Lakes Shirwa and Nyassa. 1858-1864. John Murray, London.

Smith JLB. 1950. A new dogfish from South Africa, with notes on other Chondrichthyan fishes, Journal of Natural History Series 12, 3: 34, 878-887.

Smith JLB. 1952. *Carcharinus zambezensis* Peters, 1852, with notes on other Chondrichthyan Fishes. Ann. Mag. Nat. Hist, Ser. 12 (5): 857-863.

Swann AJ. 1910. Fighting the Slave Hunters in Central Africa: A Record of Twenty-Six Years of Travel and Adventure Round the Great Lakes and of the Overthrow of Tip-Pu-Tib, Rumaliza and Other Great Slave-Traders. Frank Cass & Co. Ltd.

Tanser GH. 1975. The Guide to Rhodesia. Winchester Press, Zimbabwe.

Wallace JH. 1967. The Batoid Fishes of the East Coast of Southern Africa, Part 1: Sawfish and Guitarfish. Investigational Report No. 15. Durban: Oceanographic Research Institute.

1. *Pristis microdon*, *P. perotteti* and *P. pristis* are now all considered to be the same species, *P. pristis* (Faria et al. 2013). [↑](#footnote-ref-1)
2. The Lundi River (now known as the Rundi River) is a tributary of the Save (formerly known as the Sabi) River and the two join in the south-eastern corner of Zimbabwe, just before the border with Mozambique. [↑](#footnote-ref-2)
3. The BMNH register record notes only ‘R.Zambezi, Presented by Dr Kirk’ for 1864.2.10.5. The register record for BMNH 1982.9.13.7 notes ‘Zambezi R., Terre, 1854 [sic], Dr Kirk’, information which appears to have been transcribed directly from the information written on the specimen (the year appears to have been mis-transcribed). The latter catalogue number is thus most likely a re-registration of BMNH 1864.2.10.5, since only two specimens were recorded in the museum’s catalogue as being received from James Kirk’s Zambezi expedition (J. Maclaine pers. comm.). [↑](#footnote-ref-3)
