# Supplementary material for: Are sawfishes still present in Mozambique? A baseline ecological study
Source: PeerJ. 2017 Feb 2;5:e2950. doi: 10.7717/peerj.2950 (PMC5292025; doi:10.7717/peerj.2950)
Supplement: Appendix III — Number of interviews completed at each site. [file peerj-05-2950-s003.docx]

**Appendix III:** Number of interviews completed at each site.

| **Province** | **Place** | **n interviewees** |
| --- | --- | --- |
| MAPUTO | Maputo | 21 |
| XAI-XAI | Xai-Xai | 1 |
|  | Praia do Bilene | 1 |
|  | Zonguene | 4 |
| INHAMBANE | Inhambane | 5 |
|  | Tofo | 5 |
|  | Benguerra | 12 |
|  | Vilankulos | 8 |
| SOFALA | Beira | 14 |
| ZAMBEZIA | Nagonya | 2 |
|  | Cuassiana | 3 |
|  | Moloque | 3 |
|  | Sakone | 3 |
|  | Therrebuane | 4 |
|  | Maquaquane | 4 |
|  | Madingo | 5 |
|  | Chuabo Dembe (Quelimane) | 3 |
|  | Chinde | 11 |
|  | Luabo | 9 |
|  | Quelimane | 4 |
| NAMPULA | Sangage | 10 |
|  | Mitepene | 10 |
|  | Mazuane | 1 |
|  | Moma | 3 |
|  | Mucoroge | 5 |
| CABO DELGADO | Pemba | 7 |
|  | Natuco | 2 |
|  | Nivulatari | 1 |
|  | Fungo | 3 |
|  | Mecufi | 3 |
|  | Quirindi | 3 |
|  | Palma | 2 |
|  | Maganja (Cabo Delgado) | 2 |
|  | Milamba & Zalala (Mocimboa da Praia) | 5 |
|  | Pangane | 6 |
|  | Ibo | 7 |
|  | Quissanga | 8 |
| TOTAL |  | 200 |
|  |  |  |
