# Supplementary material for: Are sawfishes still present in Mozambique? A baseline ecological study
Source: PeerJ. 2017 Feb 2;5:e2950. doi: 10.7717/peerj.2950 (PMC5292025; doi:10.7717/peerj.2950)
Supplement: Appendix IV — Local names for sawfishes in Mozambique, by region (from south to north). Only names which were mentioned by more than one interviewee have been included. [file peerj-05-2950-s004.docx]

**Appendix IV:** Local names for sawfishes in Mozambique, by region (from south to north). Only names which were mentioned by more than one interviewee have been included.

| **Region** | **Local names** |
| --- | --- |
| Maputo | *Mbilu; salipanga* |
| Gaza province | None |
| Inhambane province | *Salpanga/ saropanga* |
| Sofala province | *Sarrapanga* |
| Zambezia province | *Piilu/ biilu; cachão/ cação; (mokuru haji*)* |
| Nampula province | *Piilu/ mpiilu/ mbilu; salipanga* |
| Cabo Delgado province | *Mbiru/ mpiru; papapanga/ papopanga; nzirué; nsungi/ papa nsungi; (mokuru-haji*)* |

*May refer to guitarfish rather than sawfish, or the name may be used for both.
