# Supplementary material for: Are sawfishes still present in Mozambique? A baseline ecological study
Source: PeerJ. 2017 Feb 2;5:e2950. doi: 10.7717/peerj.2950 (PMC5292025; doi:10.7717/peerj.2950)
Supplement: Appendix V — Current location, reported capture location, standard rostral length measurements (as defined by Whitty et al., 2013), number of rostral teeth and species identification for all sawfish rostra observed during the study and during a visit to Zambezia province in 2016. [file peerj-05-2950-s005.docx]

**Appendix V:** Current location, reported capture location, standard rostral length measurements (as defined by Whitty et al. 2013), number of rostral teeth and species identification for all sawfish rostra observed during the study and during a visit to Zambezia province in 2016.

| **Location** | **Reported location of capture** | **Owner** | **SRL (m)** | **SRW: SRL ^a^** | **N rostral teeth L/ R ^b^** | **Species** |
| --- | --- | --- | --- | --- | --- | --- |
| Maputo* | unknown | Maputo Natural History Museum | 1.24 | 0.21 | 19/ 19 | *P. pristis* |
| Maputo* | unknown | Maputo Natural History Museum | 0.75 | 0.21 | c.20/ c.21 | *P. pristis* |
| Maputo | Nampula/ Cabo Delgado provinces | Privately owned | 1.16 | 0.21 | 17/ 18 | *P. pristis* |
| Zambezia province | Zambezia province | Privately owned | 0.53 | 0.18 | 18/ 18 | *P. pristis* |
| Zambezia province | Zambezia province | Privately owned | 0.29 | 0.19 | 16/ 16 | *P. pristis* |
| Zambezia province | Zambezia province | Privately owned | 0.70 | 0.20 | 20/ 19 | *P. pristis* |
| Zambezia province | unknown | Privately owned | 0.50 | 0.19 | 20/ 19 | *P. pristis* |
| Zambezia province | Zambezia province | Privately owned | 1.09 | 0.22 | 18/ 18 | *P. pristis* |
| Zambezia province | Zambezia province | Privately owned | 0.79 | 0.21 | 21/ 21 | *P. pristis* |
| Nampula province | Zambezia province | Privately owned | n/a | n/a | Unclear ^c^ | *P. pristis* ^c^ |
| Nampula province | Nampula province | Privately owned | 0.75 | 0.19 | 20/ 20 | *P. pristis* |
| Maputo* | unknown | Maputo Natural History Museum | 0.93 | 0.12 | 31/ 30 | *P. zijsron* |
| Sofala province | Caught by trawler on Sofala Bank | Privately owned | 1.10 | 0.12 | 29/ 28 | *P. zijsron* |
| Inhambane province* | unknown | Tourist lodge | 0.91 | 0.12 | 31/ 30 | *P. zijsron* |

1. SRW:SRL ranges: 0.15 – 0.25 for *P. pristis*; 0.09 – 0.17 for *P. zijsron* (Whitty et al. 2013).
2. Rostral tooth count ranges: 16 - 24 for *P. pristis*; 24 – 31 for *P. zijsron* (Whitty et al. 2013).
3. This rostrum had a handle cut into its base, making the enumeration of rostral teeth and a definite species identification impossible.

*Capture locations were unknown and thus it was not possible to confirm that these rostra came from sawfishes caught in Mozambican waters.
